# Selective removal of ammonia from wastewater using Cu(II)-loaded Amberlite IR-120 resin and its catalytic application for removal of dyes

**DOI:** 10.1007/s11356-023-25677-3

**Published:** 2023-02-08

**Authors:** Marwa A. El-Ghobashy, Mohamed M. Khamis, Abeer S. Elsherbiny, Ibrahim A. Salem

**Affiliations:** Chemistry Department, Faculty of Science, Tanta University, Tanta, 31527 Egypt

**Keywords:** Ammonia, Amberlite IR-120, Complexation, R-Cu(II)-amine composite, Dyes, Removal

## Abstract

Cationic ligand exchange is one of the most predominant mechanisms for the removal of ammonia from wastewater through complex formation. The complexation technique occurs between the metal ions loaded on the surface of Amberlite IR-120 and ammonia which is present in the medium. Cu(II)-loaded Amberlite IR-120 (R-Cu^2+^) was prepared and described using FT-IR, TGA, SEM, and EDX techniques. The prepared R-Cu^2+^ was applied for the elimination of ammonia from an aqueous solution. Different cations such as Co^2+^ and Ni^2+^ were loaded onto Amberlite IR-120 to study the impact of counter cation on the removal efficiency of ammonia. The ammonia removal percentage followed the order; R-Cu^2+^  > R-Ni^2+^  > R-Co^2+^. The effects of contact time, pH, initial concentration, temperature, and coexisting ions on the removal of ammonia from wastewater by R-Cu^2+^ were investigated. The equilibrium adsorbed amount of ammonia was found to be 200 mg/g at pH = 8.6 and 303 K within 60 min using 0.1 g R-Cu^2+^ and an initial concentration of ammonia of 1060 mg/L. The removal of ammonia using R-Cu^2+^ obeyed the non-linear plot of both Freundlich and Langmuir isotherms. According to the thermodynamic parameters, the adsorption of ammonia onto R-Cu^2+^ was an endothermic and spontaneous process. The time-adsorption data followed the pseudo-second-order and intraparticle diffusion models. Moreover, the resulting product (R-Cu(II)-amine composite) from the adsorption process exhibited high catalytic activity and could be low-cost material for the elimination of dyes such as aniline blue (AB), methyl green (MG), and methyl violet 2B (MV2B) from wastewater.

**Graphical Abstract:**

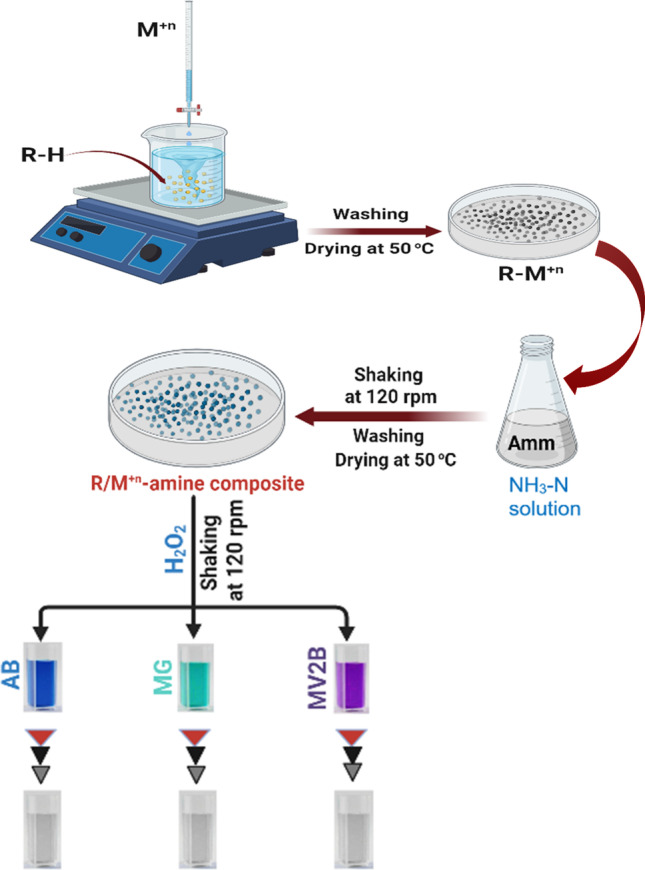

**Supplementary Information:**

The online version contains supplementary material available at 10.1007/s11356-023-25677-3.

## Introduction

Water is the most indispensable thing for life on earth. water contamination is one of the most serious issues facing humans and living organisms worldwide due to agricultural activities, industrialization, and rapid-growing global population (Elkady et al. 2020). Ammonia nitrogen (NH_3_-N) plays an essential role in marine life, it represents a nutrient for plant growth. However, it is the primary pollutant in wastewater, it results from municipal sewage, industrial wastewater, and agricultural sources or from the decomposition of organic nitrogen compounds which discharged into water streams (Zhu et al. 2019). The fertilizers industry and the manufacturing of many industrial products such as fibers, explosives, plastics, rubber, pulp, paper, chemicals, and pharmaceuticals are considered the major source of ammonia-contaminated water (Karri et al. 2018).

Large amounts of contaminated water containing high concentrations of ammonia leak into the surrounding water resources. As a result, serious environmental problems were caused such as the eutrophication of surface water due to algae growth that leads to the reduction of dissolved oxygen in water, and toxicity to fish and aquatic life (Adam et al. 2019, Zhu et al. 2019).

According to the World Health Organization (WHO) and the U.S. environmental protection agency (EPA), a level of ammonia in surface water of more than 10 mg N L^−1^ is more dangerous to human health and other aquatic organisms’ life (Liu et al. 2018). However, the maximum concentration of ammonia in drinking water and natural water should be 1.5 mg N L^−1^ and 0.2 mg L^−1^, respectively.

Nowadays, with the increasing toxic effect of ammonia on human health and aquatic organisms, the removal of ammonia (NH_4_^+^) from wastewater is the main task. There are different methods for the removal of NH_4_^+^ including chemical precipitation (Han et al. 2021), photocatalytic oxidation (Yu, Yu et al. 2021), air stripping, break-point chlorination (Zhang et al. 2022), biological methods such as nitrification and denitrification (Adam et al. 2019), membrane filtration (Rohani et al. 2021), reverse osmosis (Gui et al. 2020), adsorption (Vocciante et al. 2018), and ion exchange (Qin et al. 2020). Specifically, ion exchange and adsorption techniques are regarded as a better choice for the treatment of NH_4_^+^-contaminated wastewater. This is due to high removal efficiency, ease of application, and operation. Moreover, the adsorbents should be effectively low cost, environmentally friendly, practicable alternative properties, and favorable for commercial purposes (Elsherbiny et al. 2017; Huang et al. 2018).

A large number of adsorbents were applied for the removal of ammonia (NH_4_^+^) from wastewater, among these adsorbents various types of zeolites (Peng et al. 2017), clays, bentonite (Cheng et al. 2019), fly ash (Tang, Xu et al. 2020), activated carbon (Ghany et al. 2017), and synthetic organic resin (Han et al. 2021). However, these conventional adsorbents have low adsorption capacity and required secondary physical and chemical treatments that increase the adsorption operation costs (Chen et al. 2017a, b, c). The existence of other competing ions such as (Na^+^, K^+^, and Ca^2+^) reduces the selectivity of imitative ion exchange resins, the maximum adsorption capacity (*q*_max_) of zeolite for removal of NH_4_^+^ is 1.54 mol kg^−1^, while in the presence of Na^+^ removal efficiency decreased from 90 to 36% (Jiang et al. 2018).

Ligand exchange technology was suggested to enhance the removal efficiency of ammonia (NH_4_^+^) by ion exchange resins (Chen et al. 2017a, b, c). Cation exchange resin was loaded with a metal ion, which formed a complex with the polluted substance. The term “ligand exchange” was suggested by Helfferich in 1962, who studied the selectivity of the ion exchanger toward ammonium ion (Chen et al. 2017a, b, c).

The removal of NH_4_^+^ from wastewater by ligand exchange reactions can be represented as the following (Chen et al. 2017a, b, c): -

1 $${R}_{2}{\left[Cu{\left({H}_{2}O\right)}_{4}\right]}^{2+}+{4NH}_{3\left(aq\right)}\rightleftharpoons {R}_{2}{\left[Cu{\left({NH}_{3}\right)}_{4}\right]}^{2+}+{4H}_{2}O$$

where *R* denotes the cation exchange resin such as Amberlite IR-120.

Several transition metals such as Cu^2+^, Ni^2+^, Co^2+^, and Zn^2+^ were loaded onto cation exchange resin and served as ligand exchangers (Chen et al. 2017a, b, c; Chen et al. 2019). There is an electrostatic interaction between the active group of ion exchange resin and metal ions. A complex between the metal ion and NH_4_^+^ was formed and NH_4_^+^ replaced the aqua molecule in the solvation shell of metal (Clark and Tarpeh 2020).

The removal of NH_4_^+^ from wastewater using titanate by cation exchange process was recently studied (Zhang et al. 2021). Chen Yan et al. used Zn(II)-supported on poly ligand exchange resin for the removal of NH_4_^+^ from wastewater (Chen et al. 2019). Recent studies showed the removal of NH_4_^+^ by high silica zeolite granules regenerated with ozone (Doekhi-Bennani et al. 2021) and manganese oxides (MnOs) (Zhang et al. 2020). Moreover, iron oxide nanoparticles dispersed on zeolite by green synthesis were utilized for the removal of NH_4_^+^ from an aqueous solution (Xu et al. 2020).

Strongly cation exchange resins such as Amberlite IR-120 (R-H) are used as adsorbents for the removal of NH_4_^+^ from wastewater. R-H is sulfonated polystyrene cross-linked with divinylbenzene (Alguacil 2019). It is characterized by the gel form of an active site strongly cationic sulfonated group, which plays an essential role in the separation of charged species from an aqueous solution. R-H resin is an excellent general-purpose cation exchange resin that can be used for a wide variety of water demineralization applications because of its high surface area, non-toxicity, and microporous spherical structure.

In this work, the removal of NH_4_^+^ from wastewater by ligand exchange technique was studied. R-H was loaded with Cu(II), Ni(II), and Co(II) ions, (R-Cu^2+^, R-Ni^2+^, and R-Co^2+^), and used for the removal of NH_4_^+^ from wastewater. The removal process was carried out through ligand exchange and complexation as a promising method to increase the selectivity of the loaded resin toward the adsorption of NH_4_^+^. The effect of contact time, pH, initial ammonia concentration, temperature, and coexisting ions were examined. The use of Cu(II), Ni(II), and Co(II) in this work is attributed to their ability to form a stable and significant colored complex with ammonia which is an indication for the removal of ammonia from the solution. Afterward, the loaded R-H with copper ammonia complex was applied as a catalyst for the oxidative degradation of some organic polluted dyes. Therefore, ligand exchange resin has a dual role in the consecutive removal of NH_4_^+^ and dyes from wastewater.

## Experimental

### Materials

Strongly acidic exchange resin, Amberlite IR-120 (R-H) was purchased from Fluka (Fluka Chemie AG, CH-9470 Buchs, Switzerland). R-H is a spherical bead. It has thermal stability of about 120 °C, its total exchange capacity is 4.4 meq/g (dry) and 1.9 meq/g (wet) (H^+^-form) (1.9 mol/L). Its particle size is 0.6–0.8 mm and moisture content is 44–48% as obtained from the supplier.

Thymol (5-methyl-2-isopropylphenol) and sodium nitroprusside dihydrate (pentacyano nitrosyl ferrate II) were purchased from LANXESS AG 50,569 cologne, Germany, and used without further purification. Ammonium hydroxide (36%), sodium hypochlorite (4–5%), and acetic acid (99%) were obtained from ADWIC, Egypt. Phosphoric acid (99%) and hydrochloric acid (30%) were purchased from SDFCL, India. Sodium hydroxide, sodium carbonate, sodium hydrogen carbonate, boric acid, cobalt (II) chloride, nickel (II) chloride, and copper sulfate were obtained from Sigma-Aldrich. Hydrogen peroxide (50%, as an oxidant) was obtained from Merck, Germany, and its initial concentration was determined using potassium permanganate (Leonard 1990). Aniline blue (AB), methyl green (MG), and methyl violet 2B (MV2B) were purchased from Sigma-Aldrich. All the chemical reagents of analytical grade.

### Instrumental measurements

The UV/Vis measurements were carried out on a high-performance double-beam spectrophotometer with an electronic temperature controller (SPECORD 210 PLUS, Analytic Jena, Germany). The pH of the medium was adjusted using a pH bench meter (AD1030, Adwa, Hungary). A water shaker thermostat (Julabo D-7633 Seelbach, Germany) was used to shake the mixtures during the adsorption process. The concentration of metals was determined by an inductively coupled plasma optical emission spectrometer (ICP-OES) Optima 7000 DV with a double monochromator and a simultaneous CCD array detector (Perkin Elmer, USA). The thermogravimetric analysis (TGA) was recorded on (Shimadzu TG-50 thermal analyzer, Japan) from 30 °C up to 800 °C with a scanning rate of 10 °C min^–1^ under N_2_. FT-IR analysis was performed using (JASCO FT-IR-4100, Japan) within the wavenumber range of 4000–400 cm^−1^. Scanning electron microscope (SEM) measurements were performed with (QUANTA FEG250 microscope). The energy dispersive X-ray spectroscopy (EDX) analysis was investigated using IT100LA operating at an accelerating voltage of 20.00 keV attached to an SEM device.

### Preparation of metal ions supported on ligand exchange resin (R-M^n^.^+^)

An appropriate amount of R-H was washed repeatedly with distilled water to remove any fine particles and non-adhesive impurities, then filtrated and air-dried before use. It was regenerated with (0.1 M HCl) to increase the capacity of the exchanger and then was washed with distilled water to remove excess Cl^−^ ions which were detected by AgNO_3_ test and dried at room temperature overnight (Salem 2001).

25 g of R-H was dispersed and saturated with 500 mL of CuSO_4_ solution (0.5 M). This mixture was magnetically stirred for 24 h to achieve the equilibrium state and the resin was converted to Cu^2+^-form. Afterward, it was filtered and washed thoroughly with distilled water several times until no Cu(II) was detected in the filtrate according to ICP-OES measurements. The R-Cu^2+^ product was dried in an oven at 50 °C for 12 h. The loaded amount of Cu(II) ions onto the resin was determined by measuring the concentration of copper ions before and after the loading process using an ICP-OES spectrometer (Chen et al. 2019). R-Ni^2+^ and R-Co^2+^ were similarly prepared using the same circumstances procedure for R-Cu^2+^.

### Batch equilibrium measurements

To investigate the adsorption capacity of cationic ligand exchange resin (R-M^2+^) for the removal of NH_4_^+^ from an aqueous solution. A fixed amount of R-M^2+^ (0.1 g) was added into a series of Erlenmeyer conical flasks (100 mL) with stoppers that contain a certain volume of distilled water (19 mL). A definite volume (1 mL) of ammonia solution (3395 mg/L) was taken by micropipette and then added to each flask and all of these flasks were immediately shaken at 120 rpm in a shaker water thermostat at a controlled temperature of 30 ± 0.2 °C for a given period. The adsorption capacities of (R-M^2+^) at different time intervals were specified by determining the concentration of NH_4_^+^ that is free in the solution. The concentration of NH_4_^+^ was determined using indothymol blue method (ITB) (Zamora-Garcia et al. 2021). This method is based on the Berthelot reaction, and it is the most widely used colorimetric method for the determination of ammonia in wastewater (Lin et al. 2019). It is based on the development of the indothymol blue color formed between ammonia and thymol. The development of indothymol color occurs through two steps; the first step involves the reaction between ammonia and hypochlorite which form monochloroamine at pH 10. The second step implies the development of indothymol blue due to the reaction between thymol and monochloroamine at pH 12. This step is catalyzed by sodium nitroprusside which reduces the reaction time to 3 min (Zhu et al. 2019). The intensity of the blue color is proportional to the concentration of ammonia present in the sample. A certain amount of ammonia solution was transferred into a 25-mL volumetric flask. To this solution, 0.25 mL of sodium nitroprusside dihydrate solution (1% w/v in H_2_O), 0.25 mL of hypochlorite solution (0.5% w/v in carbonate buffer at pH 10), and 0.5 mL of thymol (6% w/v in sodium hydroxide 2 M) were added. This mixture was diluted to 25 mL with distilled water and left for 3 min. The absorbance of the blue color was recorded at *λ*_max_ = 693 nm using a UV–Vis spectrometer (SPECORD 200 PLUS) (Loan et al. 2013, Zamora-Garcia et al. 2021).

The removal efficiency percentage, *R* (%), and the adsorption capacity at time* t*, *q*_*t*_ (mg/g) of NH_4_^+^ solution onto R-M^2+^ at different times were estimated using Eqs. 2 and 3.

2 $$R\left(\mathrm{\%}\right)= \frac{{C}_{o}-{C}_{t}}{{\mathrm{C}}_{\mathrm{o}}}\times 100$$

3 $${q}_{t}= \frac{{(C}_{o}-{C}_{t}) \times V}{m}$$

where *C*_*o*_ represents the initial concentration of NH_4_^+^ (mg/L), *C*_*t*_ represents the non-adsorbed concentration of NH_4_^+^, *V* is the volume of solution (L), and *m* (g) is the mass of adsorbent.

For R-Cu^2+^ as an adsorbent, the influence of temperature on NH_4_^+^ uptake was performed at 20, 30, and 40 °C ± 0.2 °C, respectively using fixed initial NH_4_^+^ concentration and amount of R-Cu^2+^ (0.1 g). Universal buffer (consisting of a certain volume of 0.2 M NaOH solution which was poured in 100 mL of a mixture of 0.04 M acetic, 0.04 M phosphoric, and 0.04 M boric) of pH range from 2 to 12 were utilized to know the impact of pH in the removal efficiency of NH_4_^+^.

### Synthetic wastewater

Synthetic wastewater was prepared by dissolving 5 g of industrial urea fertilizer in 100 mL of distilled water. A certain amount of sodium chloride, potassium chloride, calcium chloride, and ammonium chloride (0.01 g of each one) was added to this solution. Then, 20 mL from this synthetic wastewater was added to 0.1 g of R-Cu^2+^ and the system was shaken in a shaker water thermostat at 120 rpm and 30 ± 0.2 °C for 60 min. The adsorbed amount of NH_4_^+^ was determined as described in “Batch equilibrium measurements.”

### Catalytic oxidation of dyes

The recovery and reusability of R-Cu(II)-amine composite after washing with distilled water several times and drying in an oven at 50 °C for 12 h were studied. It was used for the catalytic degradation of three dyes by the oxidation process. Stock solutions (1 × 10^−3^ mol L^−1^) of AB, (5 × 10^−4^ mol L^−1^) of MG, and (2.4 × 10^−3^ mol L^−1^) of MV2B dyes were prepared. The optimum reaction mixture was set up in a 100 mL Erlenmeyer flask. In these flasks, 0.05 g of R-Cu (II) amine composite was added to a certain volume of the dye’s solution and the appropriate concentration of H_2_O_2_ solution. The flasks were put in a water shaker thermostat at 30 °C ± 1 °C and agitated at 120 rpm for a given period. The change in the absorbance of each dye solution was determined with time.

The degradation efficiency was determined using Eq. 4.

4 $$\mathrm{Degradation}\;\mathrm{efficiency}\;\left(\%\right)\frac{A_o-A_t}{A_o}\times100$$

where *A*_*o*_ is the initial absorbance of dye and *A*_*t*_ is the absorbance at time *t* (min).

## Results and discussion

### Loading of metal ions on R-H

R-H was loaded with different amounts of copper (II), nickel (II), and cobalt (II) as measured by ICP-OES spectroscopy. It was found that 1 g of R-H has been loaded with 122.8 mg/g, (3.864 meq/g, 87.8%), 3.960 mg/g, (0.134 meq/g, 3.05%), and 0.750 mg/g (0.026 meq/g, 0.59%) of copper (II), nickel (II), and cobalt (II), respectively.

This can be attributed to the selectivity of the sulfonate (-SO_3_H) functional group of R-H towards loaded metal ions. It was written that this selectivity increases with increasing the atomic size of metal ions (Chandrasekara and Pashley 2015). The ionic radius of three metals decreases according to the order Cu(II) > Ni(II) > Co(II). As a result, the highest loaded amount on R-H was found for Cu(II).

### Characterization

#### FT-IR

**Fig. 1:**
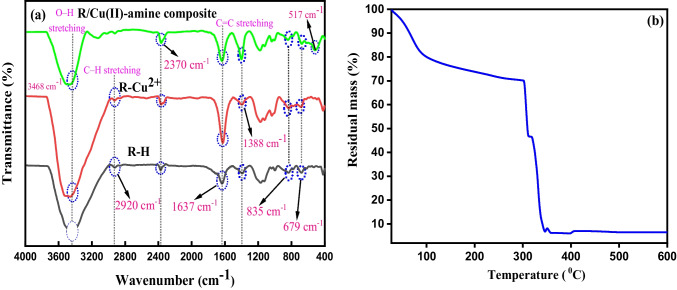
(**a**) FT-IR spectra of R-H, R-Cu^2+^, and R-Cu(II)-amine composite**;** (**b**) TGA curve of R-Cu(II)-amine composite

The FT-IR spectra of R-H and R-Cu^2+^ are shown in Fig. 1a. For parent R-H, there is a stretching vibration of O–H groups which originated from H_2_O molecule due to moisture are shown around 3434 cm^−1^. The peaks at 2920 cm^−1^ have corresponded to the symmetric and asymmetric C–H stretching vibration of C–H and –CH_2_ groups. The observed peak at 2370 cm^−1^ is due to O–H stretching mode that is originated from the polymer (Singare et al. 2011). The band at 1637 cm^−1^ indicates C = C stretching vibration of aromatic rings. Stretching vibration of O–S–O group of sulfonic acid was recorded at 1388 cm^−1^ and the peaks observed at 1170 cm^−1^ and 999 cm^−1^ are represented to symmetric stretching of –SO_3_^−^ group (Prekob et al. 2019). The band at 835 cm^−1^ represented bending C–H out-of-plane deformation of the aromatic ring and at 679 cm^−1^ attributed to –SO_3_H groups (Ghosh et al. 2015). After loading Cu^2+^ on the resin’s surface (R-Cu^2+^), the stretching –OH peak at 3434 cm^−1^ was shifted to 3460 cm^−1^ suggesting the formation of Cu(OH)_2_ (Idrees et al. 2018). The peaks at 1170 cm^−1^ and 999 cm^−1^ were shifted to 1188 cm^−1^ and 1036 cm^−1^, respectively, suggesting the formation of the coordinated covalent bond between Cu(II) and sulfonate groups (Jha et al. 2009). However, the peak at 679 cm^−1^ kept on its position as in R-H (679 cm^−1^) indicating some of –SO_3_H groups were still free on the resin surface.

#### TGA

The thermal stability of R-Cu(II)-amine composite was investigated via TGA technique as displayed in Fig. 1(b). The thermogram of R-Cu(II)-amine composite demonstrated that a total loss of weight of about 93.08% in three separate steps. The first step showed that about 19.42% loss of weight was at the temperature range of 28–100 °C. This is attributed to the evaporation of adsorbed water molecules from the surface. The second step represented the complete decomposition of amino ligands in the complex of Cu (II)–amine with a weight loss of 9.677% in the range of 100–295 °C (Inba et al 2013). The last weight loss step was noted above 295 °C with 63.96% degradation. This corresponds to the degradation of the organic polymer of R-H leaving thermally stable metal oxide as a residue (Singare et al. 2011).

#### SEM

**Fig. 2:**
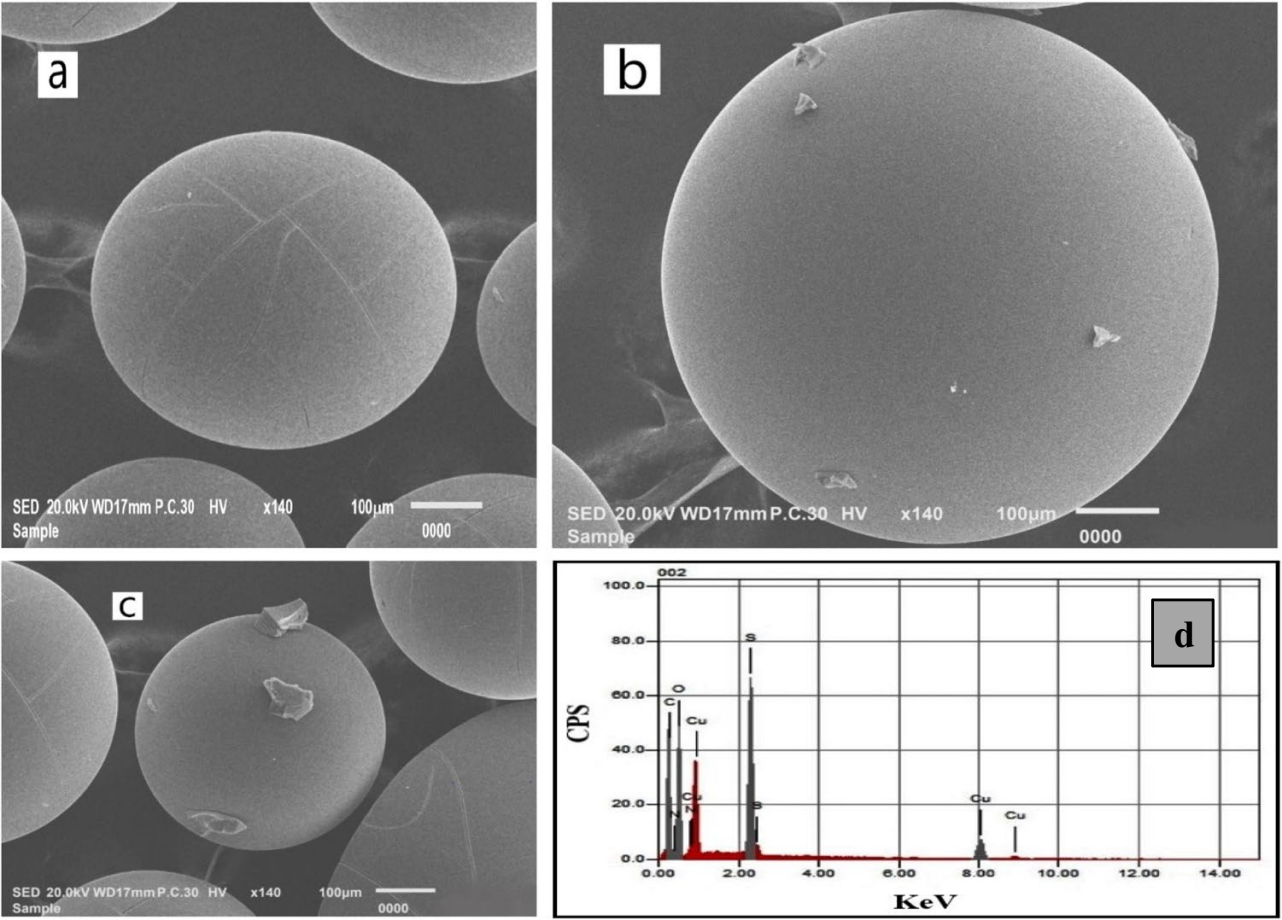
SEM image of (**a**) R-H; (**b**) R-Cu; (**c**) R-Cu(II)-amine composite; and (**d**) EDX image of R-Cu(II)-amine composite.

The surface morphology of R-H, R-Cu^2+^, and R-Cu(II)-amine complex was investigated by SEM (Fig. 2a, b, c). The surface of pure R-H is a plane spherical structure. But in the case of R-Cu^2+^, it was observed that some grains have been generated on its surface which indicates the impregnation of R-H with Cu^2+^ (Singare et al. 2011). Also, the grain species turned out to be a coarse coating at the surface of R-Cu(II)-amine composite which indicated the complex formation between ammonia and the Cu^2+^ ions at the surface of R-Cu^2+^.

#### EDX

The adsorption of ammonium ions on the surface of R-Cu^2+^ was confirmed by EDX measurement. Figure 2d presents the EDX spectra of R-Cu(II)-amine composite. EDX composition analysis for R-Cu(II)-amine complex indicates the presence of C, N, O, S, and Cu elements (Table 1).

**Table 1:** EDX composition analysis for R**-**Cu(II)-amine composite

Elements	Weight %
C	48.5
N	10.3
O	34.9
S	4.15
Cu	2.16

### Kinetics of NH_4_^+^removal by metal ions supported on ligand exchange resin (R-M^n+^)

**Fig. 3:**
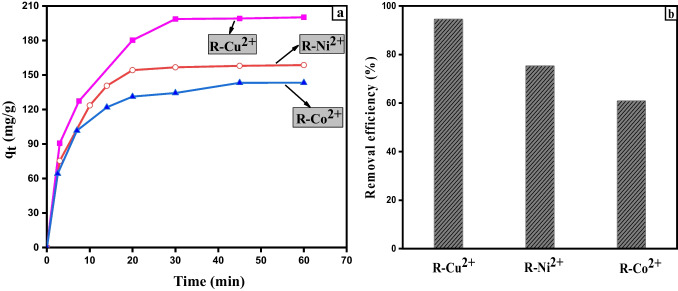
**a** Effect of contact time on the adsorption capacities of NH_4_^+^; **b** the removal efficiency of NH_4_^+^ onto R-Cu^2+^, R-Ni^2+^, and R-Co^2+^ (0.1 g) using [NH_4_^+^]_o_ = 1060 mg L^−1^, 120 rpm, and 30 °C

A comparison study between the removal efficiency of NH_4_^+^ using R-Cu^2+^, R-Ni^2+^, and R-Co^2+^, at the same initial concentration of NH_4_^+^ was carried out. It was found that the uptake amount of NH_4_^+^ by R-Cu^2+^ (*q*_*e*_ = 200 mg/g) was more than that by R-Ni^2+^ (*q*_*e*_ = 158.55 mg/g) and by R-Co^2+^ (*q*_*e*_ = 143.18 mg/g), as shown in Fig. 3a. The highest removal efficiency in the case of R-Cu^2+^ can be attributed to the highest loaded concentration of Cu^2+^ ion on the surface of R-H than Ni^2^ and Co^2**+**^ (Demirbas et al. 2005). In this work, the adsorption process was governed by two processes. One was the ion exchange between (H^+^) which is the non-loaded parts of R-H with NH_4_^+^. The other one was the metal-nitrogen bonding between the loaded Cu^2+^ and NH_4_^+^ at the surface (Clark and Tarpeh 2020) (see Fig. 8). So, R-Cu^2+^ was established as the best adsorbent which can be applied for the removal of NH_4_^+^ from wastewater. So, detailed experiments concentrated on the removal efficiency of NH_4_^+^ using R-Cu^2+^ were achieved under different conditions.

The adsorbed amount of NH_4_^+^ onto R-Cu^2+^ changes within time. It increased sharply in the first 20 min and attained the equilibrium state within 50 min (Fig. 3a).

The initial rapid adsorption of NH_4_^+^ onto R-Cu^2+^ can be assigned to a large number of the exchangeable active sites which increases both exchange and complexation’s rates. After that, the adsorption process became slower and slower till reached equilibrium due to no vacant active site on the surface. The maximum adsorption capacities (*q*_max_) of NH_4_^+^ on different loaded resins, nanocomposites, and metal oxides surfaces reported in the literature were shown in Table 2. R-Cu^2+^ has the highest adsorption capacity of NH_4_^+^ in an alkaline medium (pH = 8.6) using a small amount of R-Cu^2+^ (0.1 g) in a short contact time, only 60 min. The other loaded resins had smaller *q*_max_ than R-Cu^2+^ despite using a big amount of each resin and long contact time (reached sometimes to 24 h) as seen in Table 2. Nickel, strontium, and zirconia oxides decorated graphene oxide (NiSr–ZrO_2_/GO) had a comparable value of *q*_max_, but still, R-Cu^2+^ showed the highest one. This reflects that R-Cu^2+^ is a good candidate for the removal of NH_4_^+^ from an aqueous solution.

**Table 2:** The comparison of the maximum adsorption capacity of R-Cu^2+^ and various adsorbents for the adsorption of NH_4_^+^ reported in literature

Adsorbent	*q*_max_ (mg/g)	Conditions	References
Zn(II)-loaded resin	38.55	1 g/100 mL, pH = 9.54, 298 K for 240 min	(Chen et al. 2019)
Cu(II)-loaded chelating resin	42.74	1 g/100 mL, pH = 9.5, 288 K for 24 h	(Chen et al. 2017a, b, c)
Cu-WAER (secondary amino group)	9.30–14.79	0.1 g/15 mL, pH (5.3–7.4), 298 K Within 20–60 min	(Mahata, Chung et al. 2021)
CuN-SiO_2_ powder (primary amino group)	21.37	0.2 g/100 mL, pH = 8, 298 K within 1 min	
Synthetic manganese oxides (MnOs)	25.77	0.4 g/100 mL, pH = 7, 298 K for 24 h	(Zhang, Wang et al. 2020)
Nickel, strontium, and zirconia oxides decorated graphene oxide (NiSr–ZrO_2_/GO)	181.8	0.05 g/30 mL, pH 5–8 at room temperature, for 60 min	(Mousavi, Nabi Bidhendi et al. 2020)
Modification bentonite (Al-Tan-Bent)	5.85	2 g/L, pH = 7.5, 298 K for 60 min	(Cheng et al. 2019)
Sodium functionalized graphene oxide (GO-Na)	32.00	1 g/L, pH = 7, 303 K for 60 min	(Mirahsani, Giorgi et al. 2020)
Ball-milled bamboo biochar (BMBB)	22.90	0.05 g/30 mL, pH = 6, 298 K for 24 h	(Qin et al. 2020)
Modified zeolite (M-Zeo)	17.83	0.5 g/25 mL, pH = 8, 293 K for 24 h	(Pan, Zhang et al. 2019)
Cu(II)-loaded on Amberlite IR-120 resin (R-Cu^2+^)	200.0	0.1 g/20 mL, pH = 8.6, 303 K for 60 min	This work

Three kinetic models namely, pseudo-first-order, pseudo-second-order, and intraparticle diffusion models (Eqs. 5, 6, and 7, respectively) were applied to find out the relation between NH_4_.^+^ concentration and adsorption rate and fitting the experimental data of adsorption (Lagergren 1898) (Weber Jr and Morris 1963, Sparks 1989; Cheng et al. 2019)

5 $$\mathit{ln}\left({q}_{e}-{q}_{t}\right)=ln{q}_{e}-{k}_{1}t$$

6 $$\frac{t}{{q}_{t}}=\frac{1}{{k}_{2}{q}_{e}^{2}}+\frac{t}{{q}_{e}}$$

7 $${q}_{t}={k}_{p}{t}^{1/2}+C$$

where *q*_*e*_ and *q*_*t*_ (mg/g) are the adsorbed amount of NH_4_^+^ at equilibrium and at contact time *t*, respectively. *k*_1_ (min^−1^), *k*_2_ (g/mg min), and *k*_*p*_ (mg.g^−1^.min^1/2^) are the rate constant of pseudo-first-order, pseudo-second-order, and intraparticle diffusion, respectively. C (mg.g^-1^) is a constant term that represents the thickness of the boundary layer.

The values of kinetic parameters, the correlation coefficient, *R*^2^, and the calculated adsorption capacity (*q*_*e,*cal_) were presented in Table 3. According to this Table, the adsorption of NH_4_^+^ onto R-Cu^2+^ did not fit the pseudo-first-order model due to the low value of the correlation coefficient (*R*^2^ ≈ 0.81). In addition, the values of (*q*_*e*_,_cal_) and the experimental one (*q*_*e*_,_exp_) were not compatible. However, the calculated (*q*_*e*_,_cal_) from the pseudo-second-order equation was matched very well with the experimental one (*q*_*e*_,_exp_). Additionally, the value of (*R*^2^) was close to unity, indicating that the pseudo-second-order model is the best one to describe the adsorption of NH_4_^+^ from an aqueous solution onto the surface of R-Cu^2+^. The validity of the pseudo-second-order model to process indicates the adsorption mechanism was carried out by the chemical adsorption process (Zhang, Wang et al. 2020; Idrees et al. 2021; Özer and İmamoğlu 2022).

**Table 3:** Kinetics model’s parameters with their correlation coefficient (*R*^2^) for the adsorption of NH_4_^+^ onto R-Cu^2+^ (0.1 g) at 30 °C

[NH_4_^+^]_0_ (mg/L)	Pseudo-first-order model				Pseudo-second-order model				Intraparticle diffusion model			
	*q*_*e* (exp)_ (mg g^−1^)	*q*_*e* (cal)_ (mg g^−1^)	*k*_1_ (min^−1^)	*R*^2^	*q*_*e* (exp)_ (mg g^−1^)	*q*_*e* (cal)_ (mg g^−1^)	*k*_2_ (g mg^−1^ min^−1^)	*R*^2^	*k*_*p*1_ (mg g^−1^ min^−1/2^)	*R*^2^	*k*_*p*2_ (mg g^−1^ min^−1/2^)	*R*^2^
398.6	71.13	86.38	0.333	0.999	71.13	75.19	0.009	0.991	22.21	0.987	1.385	0.835
621.0	113.5	72.73	0.102	0.946	113.5	119.1	0.003	0.996	35.37	0.997	1.619	0.982
855.3	159.5	152.7	0.160	0.842	159.6	188.7	0.001	0.936	68.48	1.000	1.919	0.588
1060	200.1	231.3	0.139	0.968	200.1	208.3	0.002	0.997	40.08	0.999	0.268	0.707
1686	316.9	354.7	0.074	0.992	316.9	357.1	0.0002	0.984	55.77	0.999	2.095	0.758

**Fig. 4:**
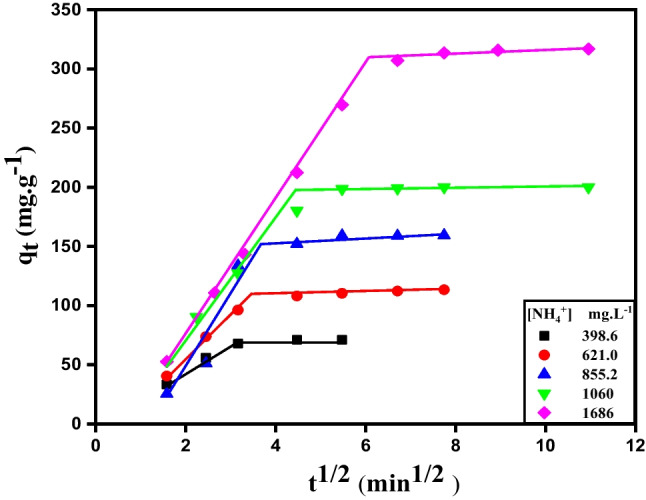
Intraparticle diffusion plot of the adsorption of NH_4_^+^ onto (0.1 g) of R-Cu^+2^ at 30 °C

The fitting data of the intraparticle diffusion model showed two straight lines as presented in Fig. 4, indicating that the adsorption mechanism involved two steps. The first step was mainly due to the film diffusion and the fitting line away from the origin implying that the adsorption of NH_4_^+^ occurred over the external surface. Therefore, intraparticle diffusion was not the rate-controlling step. The second step was assigned to intraparticle diffusion (Elsherbiny et al. 2020). The values of *k*_*p*1_ and *k*_*p*2_ were listed in Table 3, where the value of *k*_*p*1_ was greater than that of *k*_*p*2_.

### Impact of pH

**Fig. 5:**
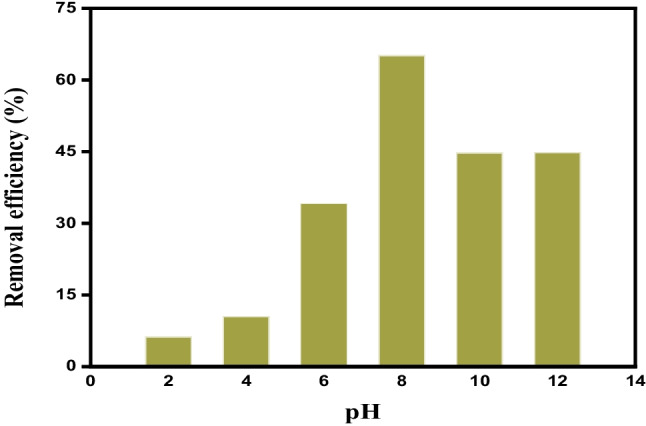
The effect of initial pH on the removal efficiency of NH_4_^+^, [NH_4_^+^]_o_ = 1032.9 mg L^−1^ using R-Cu^2+^  = 0.1 g at 30 °C

The pH of the medium is one of the most critical factors for the removal of NH_4_^+^ by R-Cu^2+^. Since it possesses a significant role in the ratio of two forms of ammonia and the adsorbent surface, the impact of pH in the removal of NH_4_^+^ was examined in the pH range from 2 to 12 using a universal buffer, while the initial concentration of NH_4_^+^, amount of R-Cu^2+^, and temperature were kept constant (Fig. 5). NH_4_^+^ in an aqueous solution is available in two forms, ammonium ion (NH_4_^+^) and unionized ammonia (NH_3_), and the proportion of both forms depends on pH and temperature. At a pH lower than 4, the amount of NH_4_^+^ uptake is slightly increased, this is due to the predominant form of ammonia in this range being NH_4_^+^. On the other hand, when the pH increases from 4 to 8, the removal efficiency increased significantly from 10.6 to 65.2%. This increase is attributed to ammonium ion being gradually converted to NH_3_ which reacts with Cu^2+^ to form Cu (II)-amine complex as shown in Eq. (1). However, above pH 8, the NH_4_^+^ removal efficiency decreased, because of the considerably increased concentration of hydroxide ions developed in an alkaline medium. This increase of OH^−^ can form a precipitate of copper (II) hydroxide Cu(OH)_2_. It was written that the formation of Cu(OH)_2_ at higher pH values leads to a decrease in the amount of copper loaded on the resin (Chen et al. 2019) and subsequently, decreases the adsorbed amount of ammonia.

### Impact of initial NH_4_^+^ concentration

**Fig. 6:**
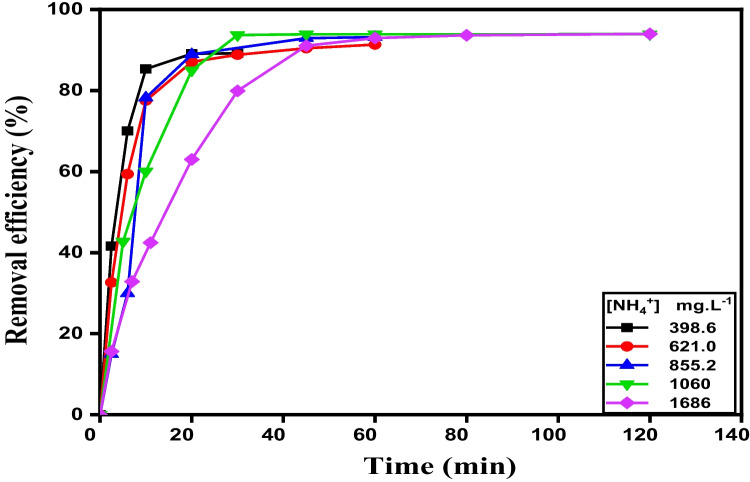
Influence of initial concentration of NH_4_^+^ solution on its removal efficiency at different time using R-Cu^2+^  = 0.1 g, pH = 8.6 at 30 °C

The impact of the initial NH_4_^+^ concentration [NH_4_^+^]_o_ was studied by changing its concentration between 398.6 to 1686 mg. L^−1^ (Fig. 6). The removal efficiency of NH_4_^+^ using R-Cu^2+^ at given time increases from 89.22 to 93.98% as the initial concentration of NH_4_^+^ raises from 398.6 to 1686 mg. L^−1^. This is due to an enhancement of the concentration gradient of NH_4_^+^ in the solution that leads to a large mass transfer driving force (Wang et al. 2020). Additionally, the available adsorption active site of adsorbent (R-Cu^2+^) becomes fewer due to continuous blocking of this site with NH_4_^+^ to form (R-Cu(II)-amine composite). On the other hand, some ammonia molecules do not get absorbed and remain free in the solution (Ding and Sartaj 2016). Moreover, the distribution coefficient, *K*_*d*_ (partition coefficient, PC) was calculated at different initial concentrations of NH_4_^+^ and its values were listed in Table 4. The partition coefficient, PC, and/or *K*_*d*_ reflect the adsorption performance of an adsorbent (Batool et al. 2020). In the case of adsorption, the partition coefficient, *K*_*d*_, is the ratio of the amount of adsorbate adsorbed per mass of adsorbent solid to the amount of the adsorbate remaining in solution per solution volume, (*K*_*d*_ = *q*_*e*_/*C*_*e*_). As seen in Table 4, the value of *K*_*d*_ is increased with increasing the initial concentration of NH_4_^+^ until 1191 mg L^−1^and after that it declined at high concentrations.

**Table 4:** Effect of the initial concentration of NH_4_^+^ on its distribution coefficient at 30 °C

[NH_4_^+^]_o_ (mgL^−1^)	*C*_*e*_ (mgL^−1^)	*q*_*e*_ (mgg^−1^)	*K*_*d*_ = *q*_*e*_/*C*_*e*_ (Lg^−1^)
398.6	42.94	71.13	1.656
621.0	53.63	113.4	2.115
855.3	57.98	159.4	2.750
1060	64.38	200.0	3.091
1119	99.97	363.9	3.639
2109	174.7	386.9	2.214
2250	206.8	408.7	1.975

### Adsorption isotherms models

**Fig. 7:**
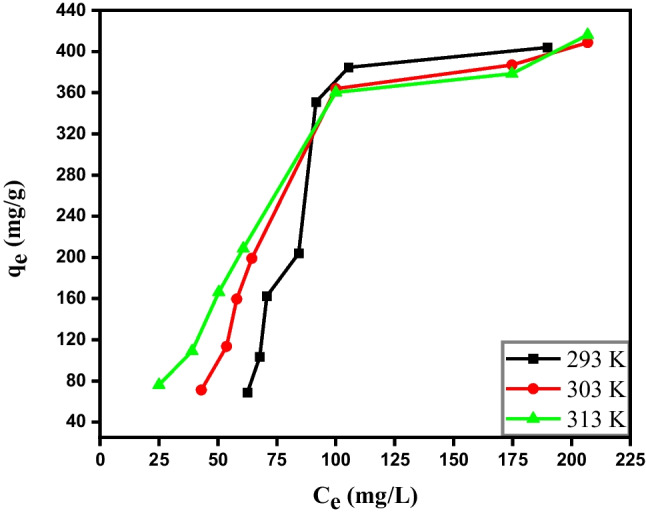
Adsorption isotherm of NH_4_^+^ by R-Cu^2+^ (0.05 g) at different three temperatures

To obtain information about the distribution of adsorbate molecules between the liquid phase and solid phase at the equilibrium state, adsorption isotherm was studied. Ammonia adsorption isotherm has been investigated at several initial concentrations and three different temperatures. Figure 7 presents the experimental isotherm data and the equilibrium adsorbed amount, *q*_*e*_ was increased as the equilibrium concentration of NH_4_^+^ raised to about 100 mg L^−1^. Afterward, no further increment was observed, and the plateau was attained. The equilibrium adsorption process was checked using linear and non-linear forms of Freundlich (Eqs. 8 and 9, respectively) and Langmuir models (Eqs. 10 and 11, respectively). Additionally, the experimental data were introduced in Temkin and Dubinin-Radushkevich (D-R) isotherms (Eqs. 12 and 13, respectively).

8 $$\mathit{ln}{q}_{e}= \frac{1}{n}\mathit{ln}{C}_{e}+\mathit{ln}{K}_{F}$$

9 $${q}_{e}={K}_{F}{C}_{e}^\frac{1}{n}$$

10 $$\frac{{C}_{e}}{{q}_{e}}=\frac{{C}_{e}}{{q}_{\mathrm{max}}} + \frac{1}{{q}_{\mathrm{max}}{ K}_{L}}$$

11 $${q}_{e}= {q}_{max} \left(\frac{{K}_{L}{C}_{e}}{1+ {K}_{L}{C}_{e}}\right)$$

12 $${q}_{e}= {B}_{1}Ln{K}_{T}+{B}_{1}Ln{C}_{e}$$

13 $$Ln{q}_{e}= {Lnq}_{m}-B{\varepsilon }^{2}$$

where *C*_*e*_ (mg L^−1^) is the concentration of NH_4_^+^ at equilibrium, *q*_max_ (mg g^−1^) is the maximum adsorption capacity of NH_4_^+^, and *K*_*L*_ (L mg^−1^) is Langmuir adsorption constant that is related to the adsorption energy (Doekhi-Bennani et al. 2021). *K*_*F*_ (mg g^−1^) and 1/*n* are Freundlich constants related to the adsorption capacity and adsorption intensity, respectively. *K*_*T*_ (L mg^−1^) is Temkin equilibrium constant connected to the maximum binding energy, and *B*_1_ (J mol^−1^) is a constant representing the heat of adsorption which is calculated from the following expression *B* = RT/*b*; *R* is the gas constant, *T* is the absolute temperature (K), and *b* is the adsorption potential (Chen et al. 2017a, b, c). *q*_*m*_ is the monolayer capacity (mg g^−1^), and *ε* is the Polanyi’s potential. The value of ε can be written as follows: *ε* = RT *ln*[1 + 1/*C*_*e*_]. The value of *B* gained from the slope of D-R’s plot is utilized to calculate the mean adsorption energy (*E*, kJ mol^−1^) which was obtained from the following equation:

14 $$E=1/{\left(-2\mathrm{B}\right)}^{0.5}$$

The linear and non-linear plots of Freundlich and Langmuir isotherms were presented in Fig. S1, S2. S3 and S4, respectively. All parameters obtained from the isotherm models along with their correlation coefficient (*R*^2^) were listed in Table 5. Visually (Figs. S1- S4) and comparing the values of (*R*^2^), the experimental data were fitted with both non-linear Freundlich and Langmuir models, especially at higher temperatures. However, the linear Langmuir model failed to represent the equilibrium adsorption data of NH_4_^+^ on R-Cu^2+^, whereas it can be described by linear Freundlich, Temkin, and D-R models. According to the data in Table 5, the values of (1/*n*) were larger than unity and decreased with increasing temperature, indicating the adsorption process becomes favorable at higher temperatures. However, the *K*_*F*_ values increase with increasing temperature. This suggests that the adsorption process is of an endothermic nature and favorable at higher temperatures (Fu et al. 2021).

**Table 5:** Isotherm parameters and the correlation coefficient, *R*^2^ for the adsorption of NH_4_^+^ onto (0.1 g) R-Cu^2+^

*T* (K)	Langmuir isotherm			Freundlich isotherm			Temkin isotherm			Dubinin-Radushkevich isotherm			
	*q*_max_ (mg/g)	*K*_*L*_ (L/mg)	*R*^2^	*K*_*F*_ (mg/g)	1/*n*	*R*^2^	*K*_*T*_ (L/mg)	*B*_1_ (J/mol)	*R*^2^	*q*_*m*_ (mg/g)	B/10^–4^ (mol^2^ J^−2^)	*E* (kJ/mol)	*R*^2^
Linear form													
293	− 595.2	− 0.003	0.104	0.264	1.475	0.651	0.023	283.9	0.963	568.4	− 13.70	19.10	0.942
303	5747	0.001	0.009	2.291	1.011	0.834	0.035	212.3	0.984	456.6	− 5.715	29.57	0.978
313	1000	0.004	0.887	8.732	0.721	0.938	0.061	152.2	0.979	394.2	− 3.109	40.10	0.986
Non-linear form													
293	2059	0.001	0.616	3.964	0.903	0.603	-	-	-	-	-	-	-
303	1068	0.003	0.858	7.580	0.762	0.835	-	-	-	-	-	-	-
313	778.9	0.005	0.933	13.419	0.654	0.902	-	-	-	-	-	-	-

From Temkin model, it was found that the value of *B*_1_ decreased with increasing temperature. In addition, the better-fitting results of Temkin model indicated that the adsorption of NH_4_^+^ is dominated by chemisorption, which is in agreement with the results of the pseudo-second-order model. The value of *E*, which was calculated form D-R model gives information about the mechanism of adsorption. Its value was higher than 20 kJ/mol (except at 293 K), indicating the adsorption of NH_4_^+^ onto R-Cu^2+^ is governed by chemical adsorption (Elsherbiny et al. 2020) coinciding with Temkin model. This value confirms that the removal of ammonia from an aqueous solution using R-Cu^2+^ was through complexation and R-Cu(II)-amine composite was formed. Additionally, this value was increased with increasing the temperature.

### Thermodynamic parameters

Deep insight into the changes in the energetic parameters related to the adsorption process was provided from thermodynamic studies. The parameters of adsorption thermodynamics can be calculated by introducing the experimental data at three different temperatures into the following equations (Elsherbiny 2013).

15 $$\mathit{ln}{K}_{d}= -\frac{\Delta {H}_{\mathrm{ads}}}{\mathrm{RT}}+\frac{{\Delta S}_{\mathrm{ads}}}{R}$$

16 $$\Delta {G}_{\mathrm{ads}}= -\mathrm{RT}\mathit{ln}{K}_{d}$$

17 $$\Delta {G}_{\mathrm{ads}}=\Delta {H}_{\mathrm{ads}}-T\Delta {S}_{\mathrm{ads}}$$

where *K*_*d*_ is the distribution coefficient (*K*_*d*_ = *q*_*e*_/C_e_), Δ*G*_ads_ is the change in Gibbs-free energy of the adsorption process, $\Delta {H}_{\mathrm{ads}}$ (kJmol^−1^) is the enthalpy change, and $\Delta {S}_{\mathrm{ads}}$ (Jmol^−1^ K^−1^) is the entropy change. The distribution coefficient (*K*_*d*_) and the thermodynamic parameters were presented in Table 6. As shown in Table 6, the value of *K*_*d*_ enhanced as temperature increased from 293 to 313 K. The values of $\Delta {S}_{\mathrm{ads}}$ and $\Delta {H}_{\mathrm{ads}}$ were determined by plotting Van’t Hoff’s equation (Eq. 15). The positive value of Δ*H*_ads_ indicates the endothermic nature of the adsorption process. While, the positive value of Δ*S*_ads_ revealed that the degree of the disorder increased at the solid–liquid interface during the adsorption of NH_4_^+^ onto R-Cu^2+^ (Pan et al. 2019). The negative values of Δ*G*_ads_ demonstrated that the adsorption of NH_4_^+^ is a feasible and spontaneous process. Furthermore, the value of Δ*G*_ads_ becomes more negative with increasing temperature, suggesting the adsorption of NH_4_^+^ onto R-Cu^2+^ was more favorable and spontaneous at higher temperatures.

**Table 6:** Thermodynamic parameters of NH^4+^ adsorbed onto (0.1 g) R-Cu^2+^

Temp (K)	*K*_*d*_ (Lg^−1^)	Δ*H*_ads_ (KJmol^−1^)	Δ*S*_ads_ (Jmol^−1^ K^−1^)	Δ*G*_ads_ (KJmol^−1^)
293	2.421			− 2.202
303	3.091	13.40	53.24	− 2.735
313	3.434			− 3.267

### Effect of competing ions

To evaluate the interference of species in industrial wastes on the adsorption process, 100 mg/L of various ions such as K^+^, Na^+^, Ca^2+^, NO_3_^−^, SO_4_^2−^, and Cl^−^ were used. The experiment was done in batches with 0.1 g of R-Cu^2+^, 1060 mg/L of ammonia, and a 60 min adsorption time. The remaining NH^4+^ concentration was measured after adsorption. The results revealed that the removal efficiency of NH^4+^ declined from 93.91 to 72.50%, 71.69%, and 48.65% in presence of Na^+^, K^+^, and Ca^2+^, respectively. This was mainly because of the competitive adsorption between cations and NH^4+^ in simulated wastewater on R-Cu^2+^ through the ion exchange process (Ren et al. 2021). So, the cations had a slight influence on the removal efficiency of ammonia except for Ca^2+^. The greatest impact of Ca^2+^ on the adsorption process is because of its high valence form (Ren et al. 2021). It was written that the selectivity of ion exchange materials is dictated by differences in ion valence, hydration radius, and hydration energy (Clark and Tarpeh 2020). Large hydration radii and high energies cause poor affinity to ion exchange functional groups: high valence causes high affinity and typically overshadows hydration effects (Epsztein et al. 2018, 2019). In terms of anions, the removal efficiency of NH^4+^ fell to 75.74%, 71.54%, and 69.48% in the existence of NO_3_^−^, Cl^−^, and SO_4_^2−^, respectively. As appeared from these results, both cations and anions have limited impact on the removal efficiency of NH^4+^ using R-Cu^2+^ because the most removal amount of NH^4+^ is due to complex formation with Cu^2+^ at the surface (Feng and Sun 2015).

### Synthetic wastewater

To study the effectiveness of R-Cu^2+^ for the removal of NH^4+^ from wastewater, an adsorption experiment was done on synthetic wastewater. The removal efficiency of NH_4_^+^ was 84.54% compared to 93.92% in absence of co-ions in water. The reason for this dropped off was the competition with the other ions in the synthetic wastewater to adsorb on R-Cu^2+^ through an ion exchange mechanism. By comparing with other studies in the literature (Soetardji et al. 2015; Mochizuki et al. 2016; Chen et al. 2017a, b, c), R-Cu^2+^ was less sensitive to the existing of competing ions towards the removal efficiency of NH^4+^.

### Adsorption mechanism

**Fig. 8:**
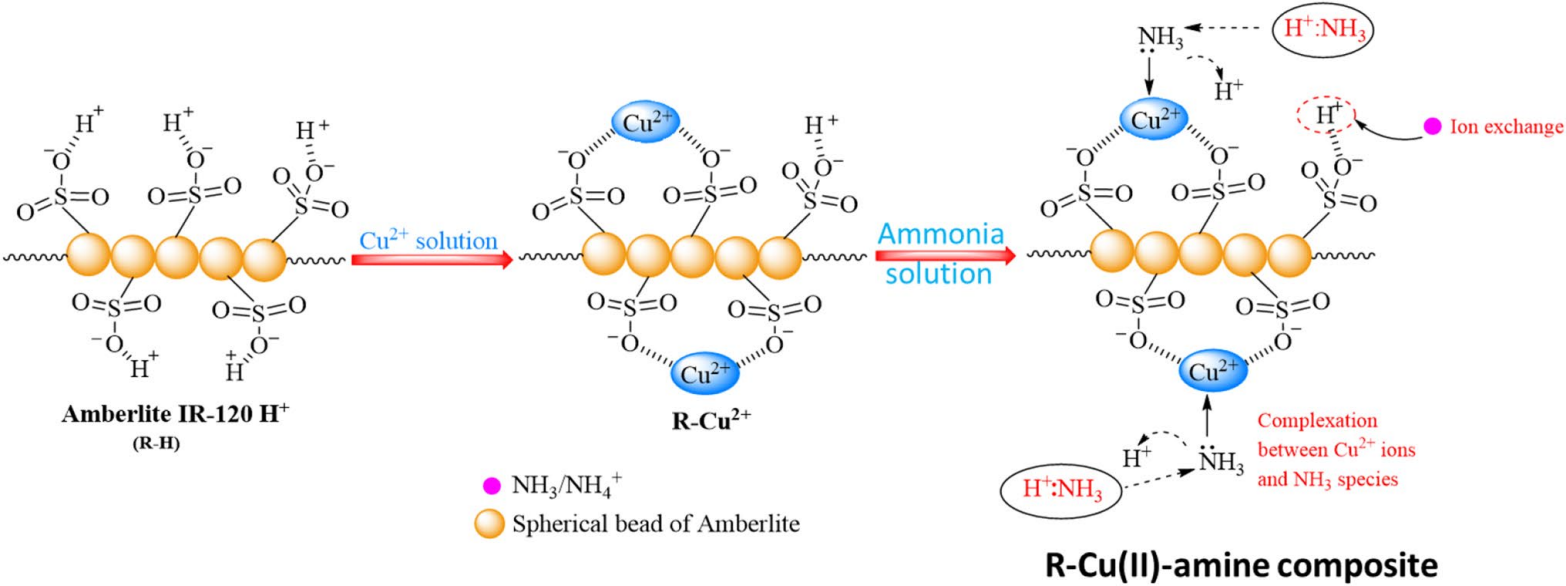
A schematic of the proposed mechanism for ammonia removal by R-Cu^2+^

The adsorption of ammonia on R-Cu(II) probably occurs through a combination of complexation and ion exchange process (see Fig. 8). FT-IR spectroscopy was used to confirm the interaction between R-Cu(II) and ammonia. FT-IR spectra of R-Cu(II) before and after the adsorption of NH^4+^ were shown in Fig. 1. After the adsorption of NH^4+^ on R-Cu(II), the peak of SO_2_ group was shifted from 1388 to 1401 cm^−1^. Moreover, a new band appeared at 517 cm^−1^ attributed to the stretching vibration of N–Cu which confirms the complex formation between Cu (II) ion and NH^4+^ (Chen et al. 2019). SEM images display the surface structure of R-Cu(II) after adsorption of NH^4+^ (Fig. 2c). It can be seen that the grain species on R-Cu(II) surface turned to be a coarse coating at the surface of R-Cu(II)-amine composite which indicated the complex formation between NH^4+^ and the Cu^2+^ ions at the surface of R-Cu^2+^. Moreover, it was written that the removal of ammonia using an ion exchanger involves the exchange of ions from the solution with ions from an exchanger (Chen et al. 2017a, b, c). So, it can be concluded that both complexation and ion exchange processes participate in the adsorption of NH^4+^ on R-Cu(II). However, complexation has the strongest role in the removal process of ammonia in this study as confirmed by the isotherm studies (“Adsorption isotherms models”).

### Catalytic activity application of R-Cu(II)-amine composite for decolorization of dyes

**Fig. 9:**
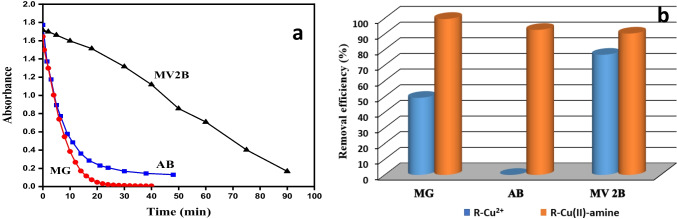
(**a**) Absorbance-time plots for the catalytic degradation of [MV] = 1.86 × 10^−4^ mol L^−1^, [AB] = 7.5 × 10^−5^ mol L^−1^, and [MG] = 2.5 × 10^−5^ mol L^−1^ using 0.05 g of R-Cu(II)-amine composite and [H_2_O_2_] = 0.01 mol L^−1^ at 30 °C. (**b**) Removal efficiency of the AB, MG, and MV2B by R-Cu^2+^ (0.05 g) and R-Cu(II)-amine composite (0.05 g) in the presence of [H_2_O_2_] = 0.01 mol L^−1^ at 30 °C

Wastewater discharge during dyes manufacturing and textile dyeing cause serious problems for both the environment and human life. This type of pollutant is produced by manufacturing poisonous and potentially carcinogenic materials, which contain highly colored organic compounds that have a low degradation ability (Salem et al. 2014). The valorization of the resulting product R-Cu(II)-amine composite was carried out. This product was applied as a catalyst to degrade three different organic dyes namely, aniline blue (AB), methyl green (MG), and methyl violet (MV2B) from an aqueous solution. The degradation of these dyes was done in the presence of H_2_O_2_ as an eco-friendly oxidant. Figure 9a presents the time decay of the absorbance of the three dyes. The degradation efficiency of each dye was calculated using Eq. (4). Figure 9b shows the degradation efficiency of the three dyes in presence of R-Cu(II)-amine composite and H_2_O_2_. At a glance at Fig. 9b, MG dye was completely degraded within 40. However, about 92.64% and 90.26% of AB and MV2B dyes were degraded after 50 min. and 90 min, respectively.

A blank experiment was done by the addition of H_2_O_2_ solution to the solution of three dyes without R-Cu(II), and R-Cu(II)-amine compost to examine the applicability of H_2_O_2_ to degrade the dyes without catalyst. No change in their colors was noticed within 4 h. On the other hand, the loaded resin R-Cu^2+^ was tested for the degradation of the same three dyes in presence of H_2_O_2_ and the results were shown in Fig. 9b. As shown in Fig. 9b, AB was not completely affected by R-Cu^2+^ as a catalyst. However, about 49.64% and 76.71% of the initial concentration of MG and MV2B were degraded, within 40 and 90 min., respectively. Comparing these results confirmed the ability of R-Cu(II)-amine composite as an efficient catalyst for the degradation of both anionic and cationic dyes from wastewater.

### Mechanism of dyes degradation

Many researchers have gone that the active species, such as hydroxyl (^•^OH) and/or perhydroxyl (HO^•^_2_), are responsible primarily for the degradation of the dye when using H_2_O_2_ as an oxidant (Salem et al. 1994; Gemeay 1996, Elsherbiny and El‐Ghamry 2015). They suggested an initial step of fast adsorption of both H_2_O_2_ and dye molecules on the surface of the catalyst (R-Cu(II)-amine). Followed by the reduction of Cu^2+^ with H_2_O_2_ to generate the less oxidative HO_2_^•^ radicals (Eq. 18). After that, Cu^2+^ will be regenerated with the formation of the hydroxyl radicals ^•^OH and OH^−^ (Etaiw et al. 2011) (Eq. 19). The generated radical species ^•^OH will attack rapidly dye molecules (Eq. 20) to form the final oxidation products as follows:

18 $$R-Cu\left(II\right)-amine+{H}_{2}{O}_{2}\stackrel{K}{\iff } R-Cu\left(I\right)-amine+{HO}_{2}^{\bullet }+{H}^{+}$$

19 $$R-Cu\left(I\right)-amine+{H}_{2}{O}_{2}\stackrel{{k}_{1}}{\to }R-Cu\left(II\right)-amine+{OH}^{-}{+}^{\bullet }OH$$

20 $$Dyes+^\bullet OH{}\stackrel{{k}_{2}}{\to }degradation\;products$$

### Recycling of R-Cu(II)-amine composite as a catalyst

**Fig. 10:**
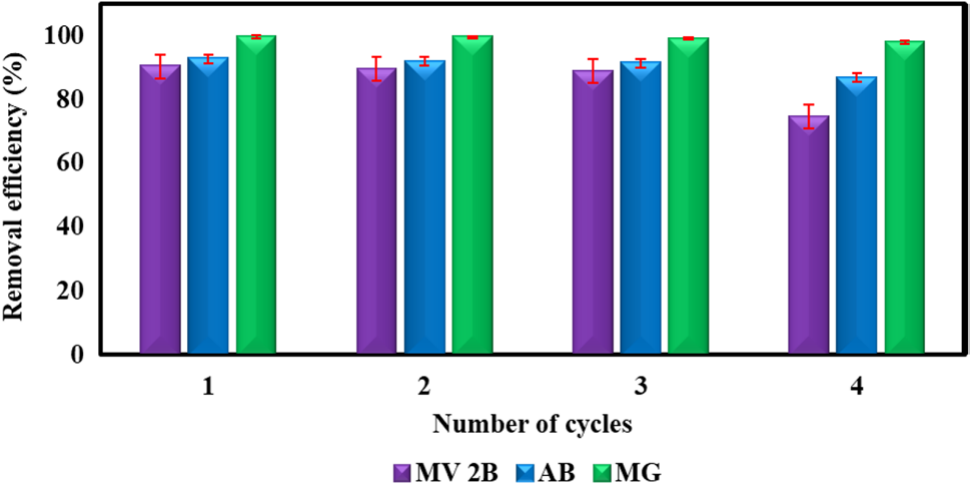
Reusability of R-Cu(II)-amine composite for the oxidation of three dyes (AB, MG, and MV2B) with H_2_O_2_

The reusability of the catalyst is fundamental to sustainable and economical operation. Here, the degradation of the three dyes (AB, MG, and MV2B) with H_2_O_2_ in the presence of R-Cu(II)-amine composite as a catalyst was carried out for four reusability cycles. At the end of each cycle, the catalyst was washed thoroughly with water and H_2_O_2_ solution, dried, and then re-entered into the next catalytic reaction cycle. This process was carried out under the same reaction conditions. Figure 10 shows that the efficiency of degradation of the three dyes with R-Cu(II)-amine composite decreased slightly through the four cycles. This confirms that this catalyst could be recycled successfully at various times with only a slight decay in its activity.

## Conclusion

In conclusion, Amberlite IR-120 (R-H) was successfully loaded with Cu^2+^, Ni^2+^, and Co^2+^ through a cation exchange mechanism to form R-M^n+^. The highest loaded amount was found in the case of Cu^2+^ with a value of 122.8 mgg^−1^ for 1 g of R-H. The loaded resin R-M^n+^ was applied to get rid of ammonia from the aqueous solution and the highest removal efficiency was found in the case of R-Cu^2+^. The presence of co-ions in the wastewater had a little negative effect on the removal efficiency of NH_4_^+^. The adsorption of NH_4_^+^ from an aqueous solution onto the surface of R-Cu^2+^ obeyed the pseudo-second-order kinetic model. The non-linear plot of Freundlich and Langmuir isotherms represented the adsorption process. The removal mechanism was governed by chemical adsorption and formation of R-Cu(II)-amine composite. The formed R-Cu(II)-amine composite was used as an efficient catalyst for the degradation of aniline blue, methyl green, and methyl violet dyes from an aqueous solution in presence of H_2_O_2_.

### Supplementary Information

Below is the link to the electronic supplementary material.

Supplementary file1 (DOCX 355 KB)

## Data Availability

All the data and materials are available in the manuscript.
