# Supplementary material for: Selective removal of ammonia from wastewater using Cu(II)-loaded Amberlite IR-120 resin and its catalytic application for removal of dyes
Source: Environ Sci Pollut Res Int. 2023 Feb 8;30(49):106822–37. doi: 10.1007/s11356-023-25677-3 (PMC10611889; doi:10.1007/s11356-023-25677-3)
Supplement: Supplementary file 1 — Supplementary file1 (DOCX 355 KB) [file 11356_2023_25677_MOESM1_ESM.docx]

**Selective removal of ammonia from wastewater using Cu(II)-loaded Amberlite IR-120 resin and its catalytic application for removal of dyes**

*Marwa A. El-Ghobashy*, Mohamed M. Khamis, Abeer S. Elsherbiny*, Ibrahim A. Salem*

*Chemistry Department, Faculty of Science, Tanta University, Tanta 31527, Egypt*

Fig. S1. Linear plot of Freundlich isotherm.

Fig. S2. Linear plot of Langmuir isotherm.

Fig. S3. Non-linear plot of Freundlich isotherm.

Fig. S4. Non-linear plot of Langmuir isotherm.
